# Indents

**Published:** 1917-01

**Authors:** 


### INDENTS

**☞ Brief Articles of Technical or Scientific Value will receive notice and comment. Contributions invited.**

*So to Speak.* One afternoon a stranger debarked from a train at a hustling town in the West and headed up the street. Finally he met a party that looked like a native.

"Pardon me," said the stranger, halting the likely looking party. "Are you a resident of this town?"

"Yes, sir," was the ready rejoinder of the other. "Been here something like fifty years. What can I do for you?"

"I am looking for a criminal lawyer," responded the stranger. "Have you one here?"

"Well," reflectively answered the native, "we think we have, but we can't prove it on him."—*Chicago Herald.*

*Repairing an Inlay Margin.* Sometimes for good and sufficient reasons it becomes necessary to seat an inlay that is good in every respect excepting a slight flaw or fault in one of the margins. This can very easily be fixed with a little gold foil. Have the surface of the inlay clean, grasp it firmly in a pair of tweezers, flux slightly and heat in a blue flame to a dull red. While at his heat a small pellet of uncondensed foil is stuck to the part of the margin this is shy. Then the inlay is placed in the cavity, allowing the foil to condence against the cavity wall. When the inlay has been seated with cement the margins will be without the former flaw.—*Pacific Gazette.*

All gold inlays should be cemented with Synthetic Cement of proper shade to correspond to the natural tooth.

If there should be a flaw or misfit in the inlay the silicate cement remedies this and will not wash out as does oxy-phosphate cement.—Burton Lee Thorpe.

*Cast Gold Crown.* Remove all decay, leaving cavities shaped as for an inlay, with the thought that this must draw and draw easily. Then remove the enamel of the tooth in whatever way seems best, in each particular case, to a line under the free margin of the gum, to where you wish your finished crown to seat. This line should be the greatest circumference of the tooth, which is covered by the crown, or in other words, form the tooth to a peg shape, cutting off the point of the peg and making it flat.

Make the band as for a shell crown, fitting it to the lines where it is seated, under the free margin of the gum, taking care that it covers no portion that was not prepared. Then place band in position on root and proceed to test the fit of the band with modeling compound. By forcing the compound while soft down into the gold band, the compound will force out wherever it does not fit. Refit until compound will not force out. Then shorten the band for articulation and give it the proper contour and points of contact with adjacent teeth. Clean it free from all compound, place it back in proper position on the root; then heat inlay wax and pack full with a hot instrument, taking great care to fill every particle of space to its extreme depth. Then place excess of wax on top of crown and have patient bite for articulation. Then carve cusps and add wax for contour, wherever needed. It is now ready to cast. Although, it is possible to make a perfectly fitting shell crown by the usual process, it may be spoiled in the setting, which is impossible with this crown.—Dr. Lee W. Atkinson, Salem, Ohio.

*Why I am Making Good.* I believe in my work.

I have prepared myself carefully for it, and am improving myself, every day, by study and observation.

I take several journals, read the new books, attend meetings and keep a careful record of my cases.

I try to be thorough, approaching every case as if it were a mathematical problem which can be solved only when I know the value of $x$ and $y$.

I take no thing and no man merely "for granted"; "search farther" is my motto—and I am looking in out-of-the-way places and books constantly for things that will help me to help my people.

I do not sulk, knock, welch or complain, but keep pleasant, keep smiling, trying to be, as well as seem to be, a friend to every man, woman and child in my town.

I am neat in my personal appearance. My nails are never "in mourning," my teeth are always white, my hands always clean, and my clothing scrupulously neat.

I am ambitious. I am determined to do better work next year than this year, and to have a better and more lucrative practice sometime—perhaps in a better town.

I am square, and my people know it—but my life advertises this fact, not my words.

I am modest, but honestly anxious to know people and to have them know me, and for that reason I am called "a good mixer." I have found that it pays to be friendly with folk, to get acquainted with them, not only because it brings me business, but also because it warms my heart and makes me a happier man.

Now you know why I am making good.

*Pain in the Feet* This troublesome subject has recently been discussed by Frauenthal. He premises the diagnosis of the cause of pain in the foot by the statement that when the pain comes on acutely it is due either to an injury or to an infection; when the pain comes on gradually, there is some relaxation of ligaments or supporting structures. He deplores the promiscuous use of foot plates, some of these metal supports doing more harm than good. A proper fitting shoe is the first essential to comfort of the foot. An arch may not be broken or may not have fallen, and may not need a support. All it needs is a proper adjustment of the heel to the foot tread and a proper arch to the shoe. Also, pain in the forward part of the foot is often not caused by a weakened arch, and an arch support will not correct pain in other weakened joints or ligaments. On the other hand, when the arch has fallen or has become weak, and there is a rotation of the ankle inward, besides a proper fitting shoe a plate or support that fits the foot and corrects the deformity may and will often entirely relieve the pain in the foot and the associated pain of the leg and back. Various exercises of the foot muscles may strengthen the arch and later allow of the removal of this artificial support.

Barnes of Washington calls attention to an overlooked cause of sore feet, and especially to the overlooked condition causing a painful ball of the foot. Pain and tenderness in this region of the foot has become much more common than formerly on account of the craze for dancing. He finds that a few anatomists call attention to sesamoid bones as being normal in this region of the foot and especially likely to be present at the metatarsophalangeal joint of the great toe, namely, the ball of the foot.

By a series of Roentgenograms of normal feet and feet that are painful in this region, Barnes finds that the sesamoid bone is part of nature's method of protecting this joint and causing normal tread and normal support. He also finds that this sesamoid bone may be fractured or may become injured and not only cause pain from its own disturbance, but cause pain in the joint above it. He orders shoes made with a depression in this region of the sole, and causes comfort, and cures his patients of their disability. In acute injuries of this region nothing will be of so much benefit as rest.

At times irritation or injury may cause an exotosis to occur in this region, which of course can be cured only by surgery.—*Journal A. M. A.*, March 18, 1916.

*The Automobile as a Cause of Poliomyelitis.* The newspapers have given an unusual amount of space to the recent epidemic of poliomyelitis, which is remarkable in view of the small number of cases of this disease as compared with epidemics of such diseases as scarlet fever, diptheria and other exanthems. An explanation is to be found in the insidious nature of the disease and its serious after-effects, in addition to the high mortality. Naturally there has been a large crop of sensational statements as to its causation and treatment. Not the least remarkable is one which has gone the rounds of the press during the past week. This paragraph reports a statement to the effect that the epidemicity of poliomyelitis is due to the fumes produced by automobiles. As the announcement is alleged to have been made before the American Public Health Association, public opinion may possibly attach undue importance to it under the impression that it is endorsed by that association. Of course to maintain such a theory it would be necessary to show that epidemics of poliomyelitis did not occur before the development of automobiles, and that they do not occur now in places in which there is little or no such development. Since 1841, when the first epidemic of this disease was reported, though not recognized, in Louisiana, until the present day, a number of these outbreaks have occurred. It will be necessary to mention only some which are inconsistent with the theory advanced in the statement referred to. Such are an epidemic of forty-three cases in 1877 at Stockholm; an epidemic of from 150 to 160 cases in the summer of 1894, in Otter Creek Valley, Vt., and an outbreak in 1899 in Norway. All of these occurred before the development of the automobile industry. In 1905 and 1906, epidemics occurred in Norway with a total of 1,053 cases, especially prevelant in the northern part of Norway. No one will allege that the northern parts of Norway even now, ten years later, are places where poliomyelitis could, by any twist of the imagination, be attributed to "gases and fumes given off in the atmosphere by the combustion of oils and fluids used in automobiles." *Post hoc, ergo propter hoc* is a familiar fallacy; *ante hoc sed propter hoc* is as novel as it is startling.

